# Supplementary material for: Naked mole-rats have distinctive cardiometabolic and genetic adaptations to their underground low-oxygen lifestyles
Source: Nat Commun. 2024 Mar 27;15:2204. doi: 10.1038/s41467-024-46470-x (PMC10973426; doi:10.1038/s41467-024-46470-x)

Supplementary Table

Species	*Common name*	*This study*			*Heart weight (mg)*	*Heart weight/ body weight*	*Maximum lifespan (years)*[1]	*Soil Type*
		Sample size	*Body weight (g)*	*Age (years)*				
Heterocephalus glaber	Naked mole-rat	n=14 (7m, 7f)	32.9±4.8	5	178.0±33.6	4.4±0.6	>37	Hard clays
Georychus capensis	Cape mole-rat	n=8 (2m, 6f)	154±16.9	5	571.7±63.1	3.3±0.2	11	Predominantly soft clays but sometimes loams
Bathyergus suillus	Cape-dune mole-rat	n=7 (5m, 2f)	639±59.8	4-5	1642.5±219.0	2.8±0.3	>6	Loose coarse sands
Cryptomys hottentotus hottentotus	Common mole-rat	n=8 (6m, 2f)	84.2±6.9	4-5	343.9±51.7	4.0±0.3	11	Hard clays and loams
Cryptomys hottentotus pretoriae	Highveld mole-rat	n=8 (3m, 5f)	102.7±11.1	4-5	559.8±50.9	4.7±0.3	11	Hard clays and loams
Cryptomys hottentotus mahali	Mahali mole-rat	n=5 (1m, 4f)	114.8±8.6	4	N/A	N/A	11	Hard clays and loams
Cryptomys hottentotus natalenesis	Natal mole-rat	n=9 (3m, 6f)	82.3±6.7	4	469.9±13.2	4.3±0.0 4	11	Compacted thick clays
Fukomys damarensis	Damaraland mole-rat	n=11 (3m, 8f)	124.6±8.2	6-7	583.3±32.6	4.3±0.3	16	Soft coarse sands and compacted fine kalahari sands
Amblysomus hottentotus	Hottentot golden mole	n=3 (1m, 2f)	58.6±8.6	Adult (>1year)	N/A	N/A	>1	Compacted thick clays
Mus musculus	*C57/BL6 mouse*	n=5 (5m)	25.0±0.1	0.1	155.3±8.7	6.2±0.3	2-3	N/A

Table S1. Morphological and life history characteristics of the taxa included in this study. m=male, f=female; N/A data not available.

Supplementary Figures

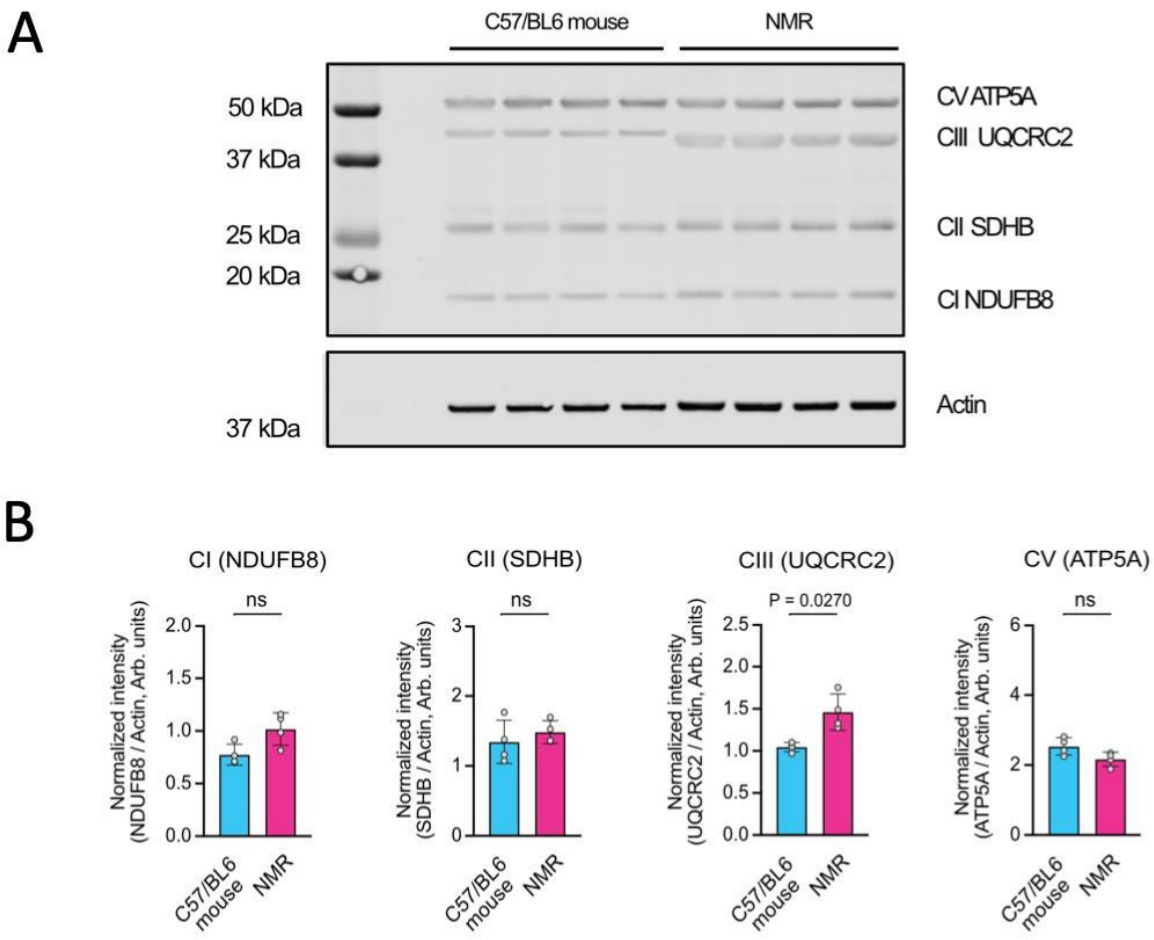

Fig. S1. Representative western blots (n = 4 animals/group) for the assessment of protein expression of mitochondrial electron transport chain (ETC) complexes, lactate dehydrogenase (LDH) and monocarboxylate transporter 1 (MCT-1) (**A**) Western blot of indicative subunits (ATP5A, UQCRC2, SDHB and NDUFB8) of mitochondrial ETC protein complexes (Complex I, II, III, and V) isolated from C57/BL6 mice and NMR hearts. (**B**) Quantification of subunits of oxidative phosphorylation complexes in C57/BL6 and NMR hearts. All experimental groups contain 4 animals per group and western blot analysis was performed using several technical replicates; protein expression normalised to loading control (details on y axis). Statistical analysis was performed using unpaired two-tailed t test (with Welch's correction). Data mean±SEM.

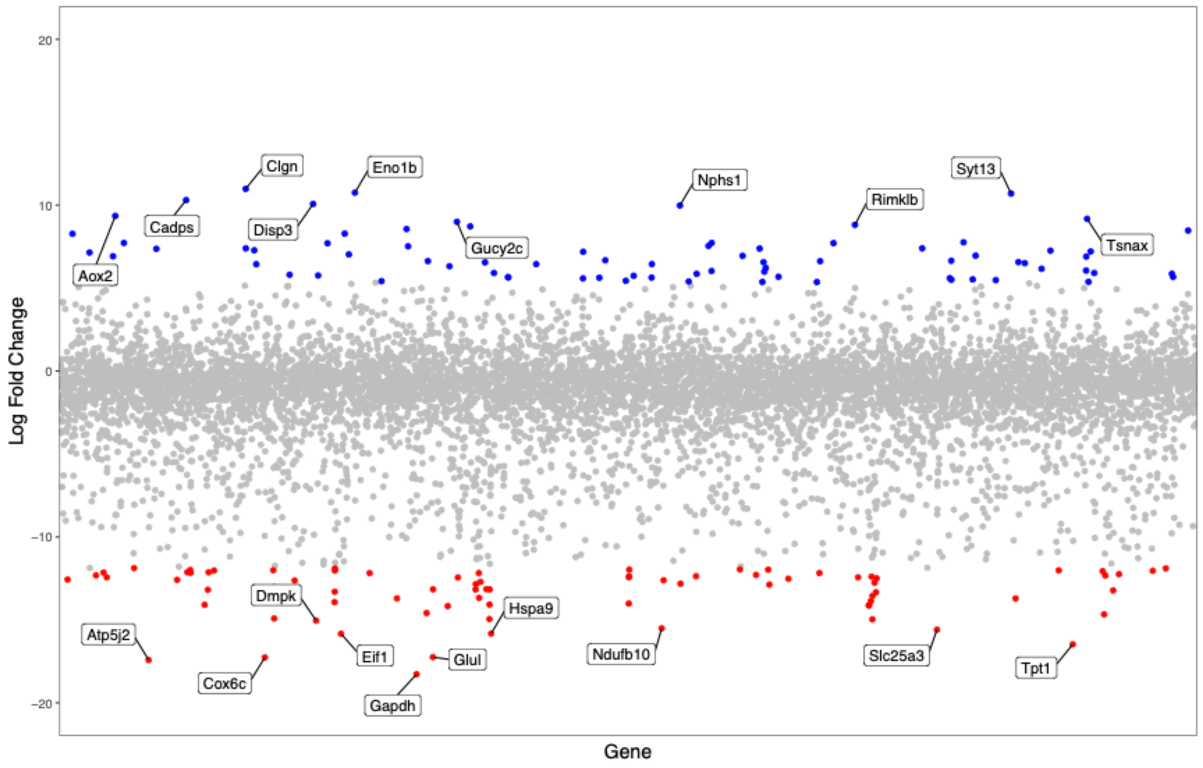

Fig. S2. Manhattan plot Log fold change gene expression NMR vs C57/BL6 Manhattan plot with top 1% gene expressed highlighted and top 10 genes labelled by name (blue for the NMR and red for the mouse). Note that of the top 20 expressed genes in the NMR, only FOUR are also expressed in the mouse and NONE of the top 12 in the NMR are also expressed in the mouse. The gene "clgn" has the highest logfold change but it is rank 1760 in genes ordered by expression (FPKM) in the NMR.

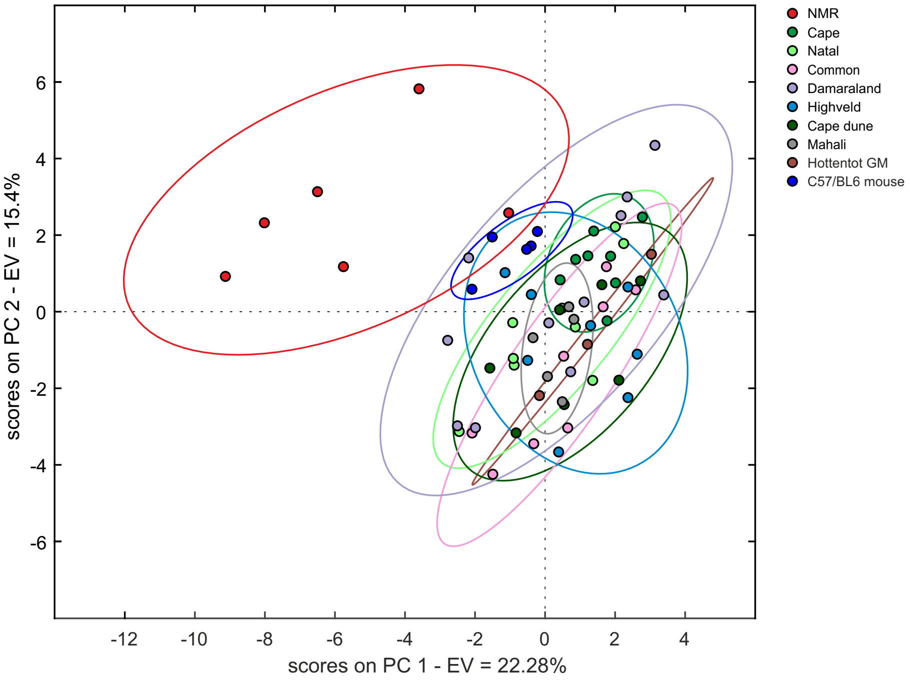

Fig. S3. Principal Component Analysis (PCA) of ^{1}H nuclear magnetic resonance metabolomic data showing separation of the NMR metabolomics data compared to all other genera. NMR (n = 6), Cape mole-rat (n = 8), Natal mole-rat (n = 9), Common mole-rat (n = 8), Damaraland mole-rat (n = 11), Highveld mole-rat (n = 8), Cape dune mole-rat (n = 7), Mahali mole-rat (n = 5), Hottentot golden mole (n = 3) and C57/BL6 mouse (n = 5), where n correspond to biological repeats. Ellipses correspond to 90% confidence intervals.

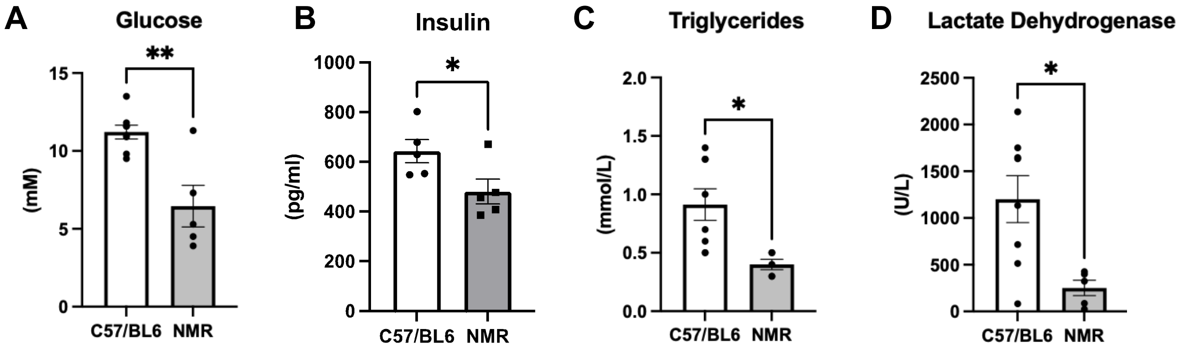

Fig. S4. Plasma metabolite concentrations of (**A**) glucose, (**B**) insulin, (**C**) triglycerides and (**D**) lactate dehydrogenase in NMR versus C57BL6 control mouse (glucose, triglycerides, lactate dehydrogenase showing reduced metabolite concentrations (NMR, n = 4 animals / group; C57BL6 n = 8 animals / group, Insulin n=5/group), data mean ± SEM, *P<0.05, ** P<0.01 two-tailed t test. Glucose P=0.0019, insulin P=0.04, triglycerides P=0.014, lactate dehydrogenase P=0.014

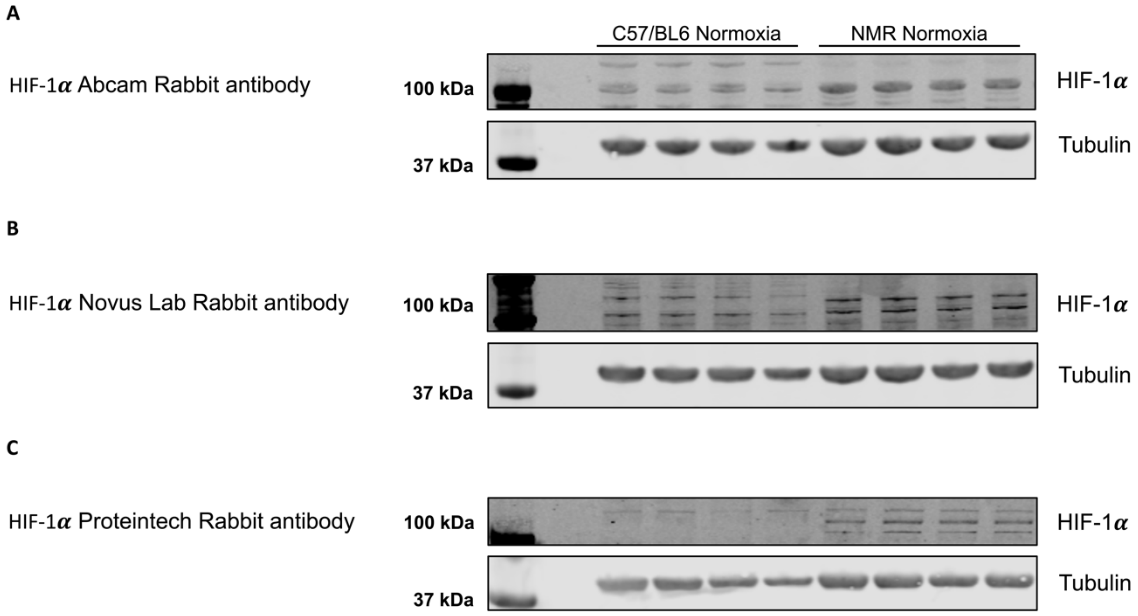

Fig. S5. Representative HIF-1α western blots using three different antibodies against different HIF-1α protein epitopes in cardiac tissue from C57/BL6 mouse and NMR (n = 4 animals / group). (**A**) Hif1α Abcam rabbit Ab, (**B**) Hif1α Novus lab rabbit Ab, (**C**) Hif1α Proteintech rabbit Ab. Uncropped blots provided at the end of the document.

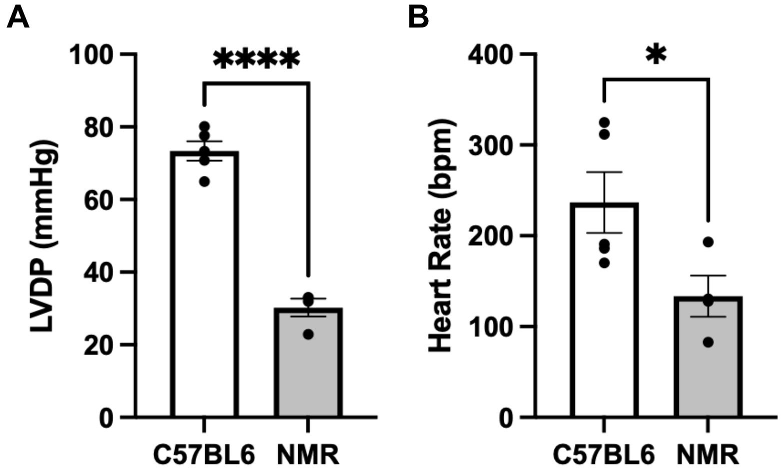

Fig. S6. *Ex vivo* cardiac function in Langedorff perfused hearts of NMR (n = 4 animals / group) and C57BL6 mouse (n = 5 animals / group), (**A**) left ventricular developed pressure (LVDP, mmHg) and (**B**) heart rate (HR, bpm), *** P=0.0001 *P=0.047 vs control by two-tailed t test.

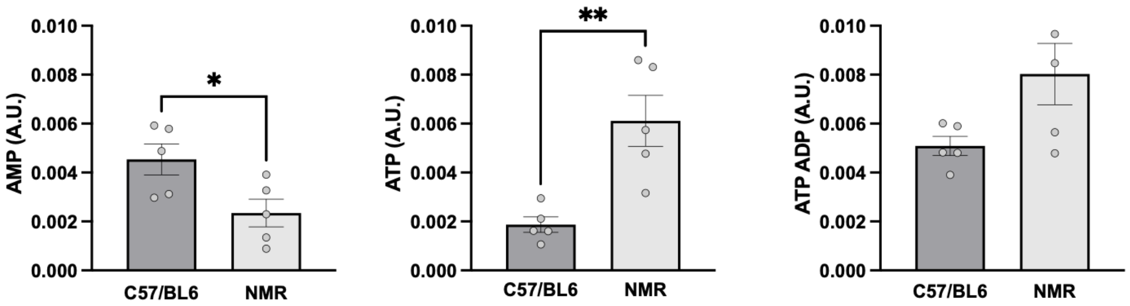

Fig S7. Individual components of myocardial total adenine nucleotide pool in post-ischemia. (n=5/group), Data mean + SEM, * P<0.05 ** P<0.01 by two-tailed t test. AMP P=0.0328, ATP P=0.0045, ATPADP P=0.055

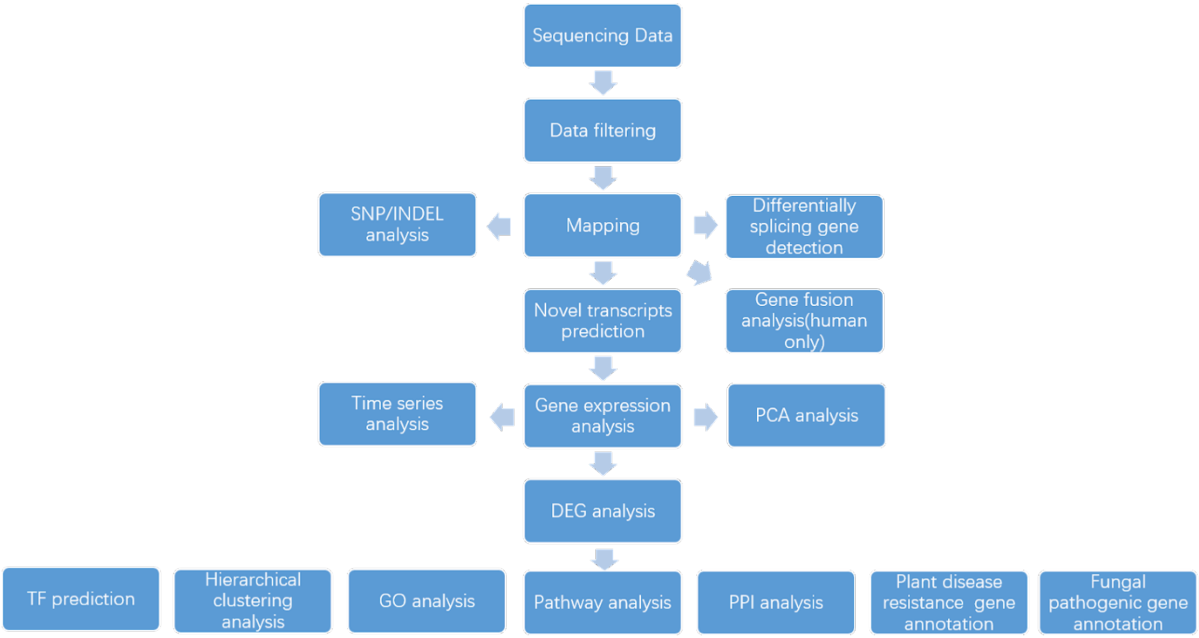

Fig S8. Summary of the bioinformatics workflow.

Supplementary Reference

1. Tacutu, Robi et al. "Human Ageing Genomic Resources: new and updated databases." *Nucleic acids research* vol. 46,D1 (2018): D1083-D1090. doi:10.1093/nar/gkx1042

Uncropped gels of blots supplied in Supplementary Figures

OXPHOS (Figure S1)

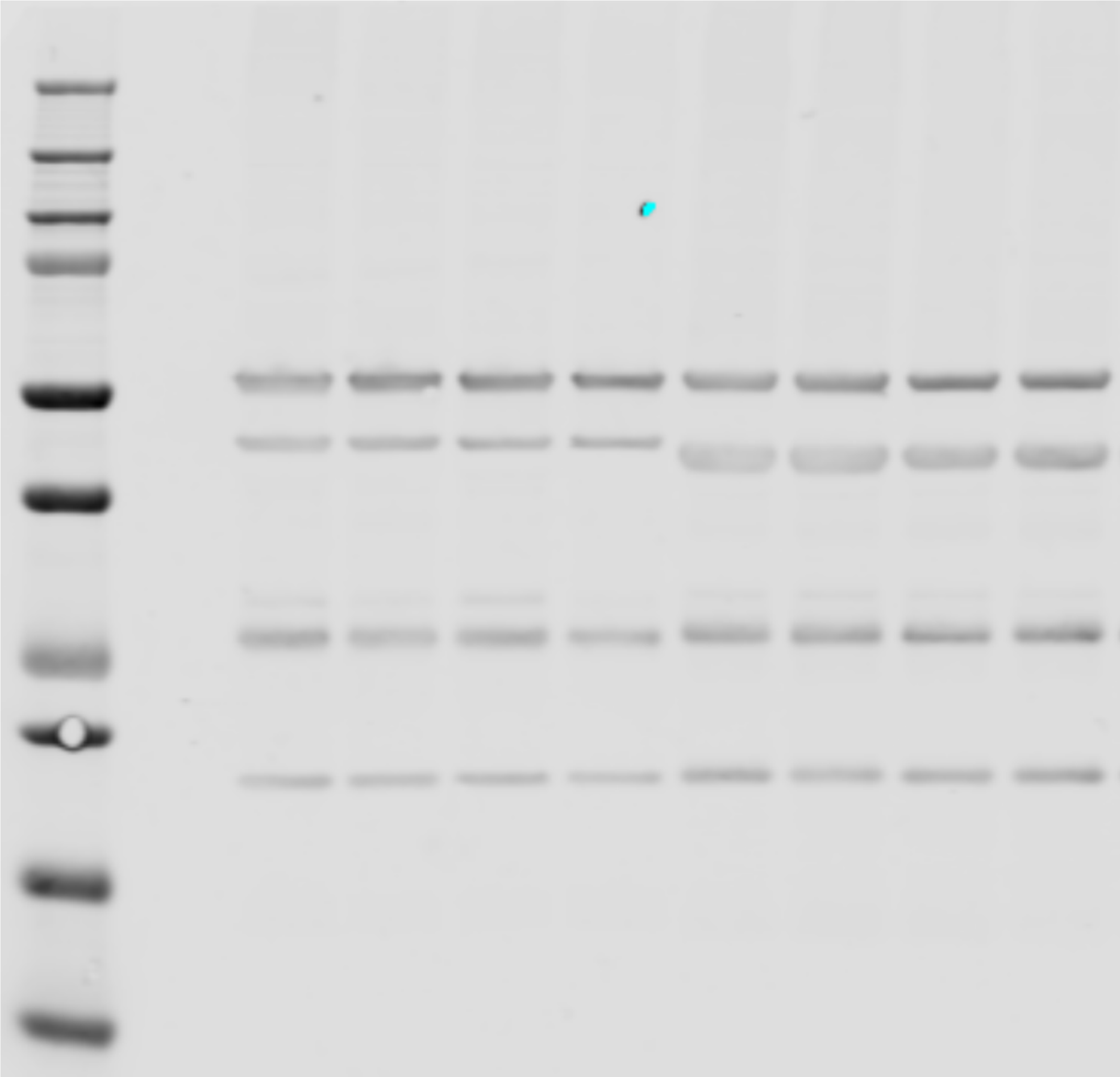

HIF1α Proteintech Antibody (Figure S5)

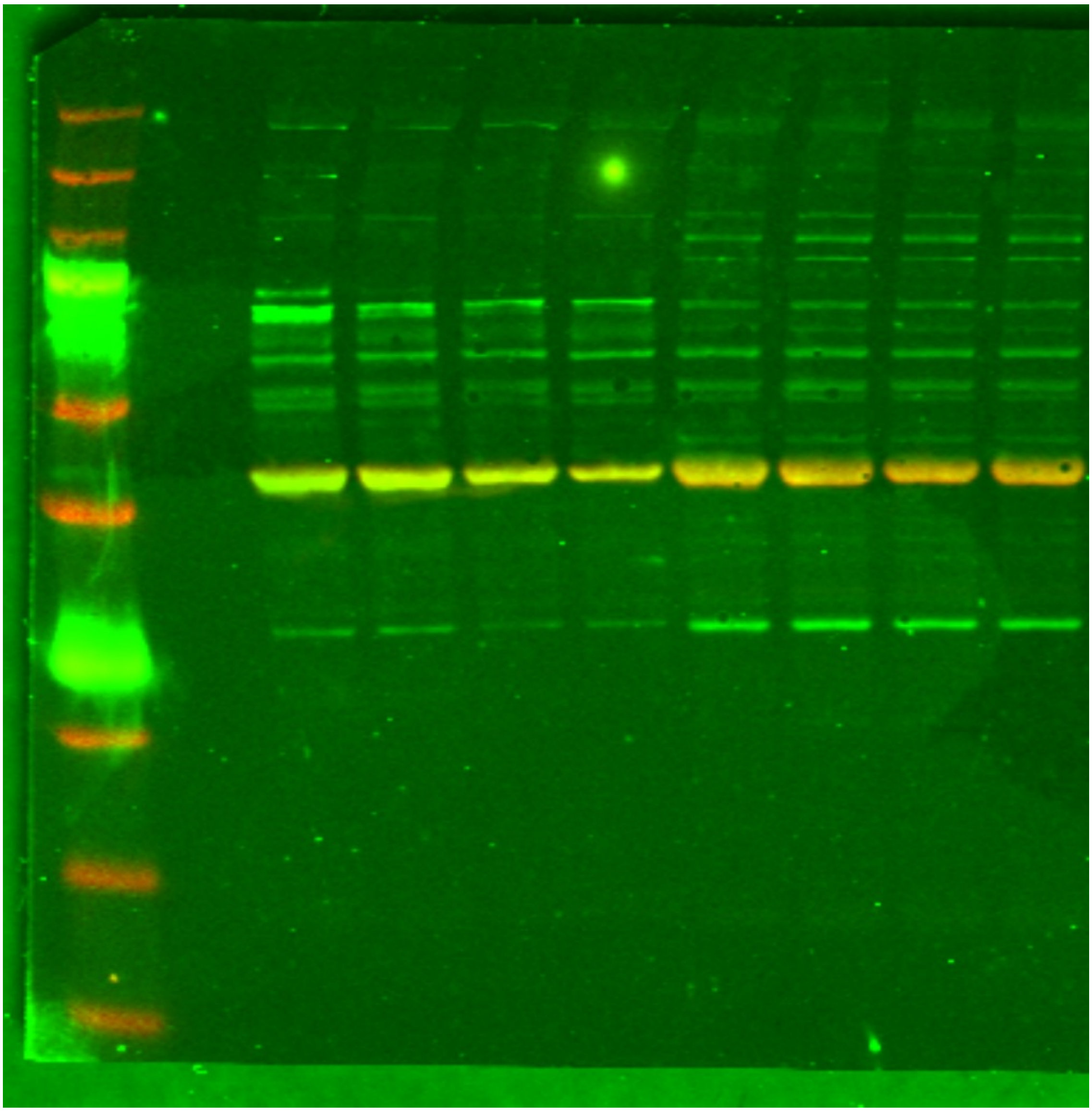

HIF1α Abcam Antibody (Figure S5)

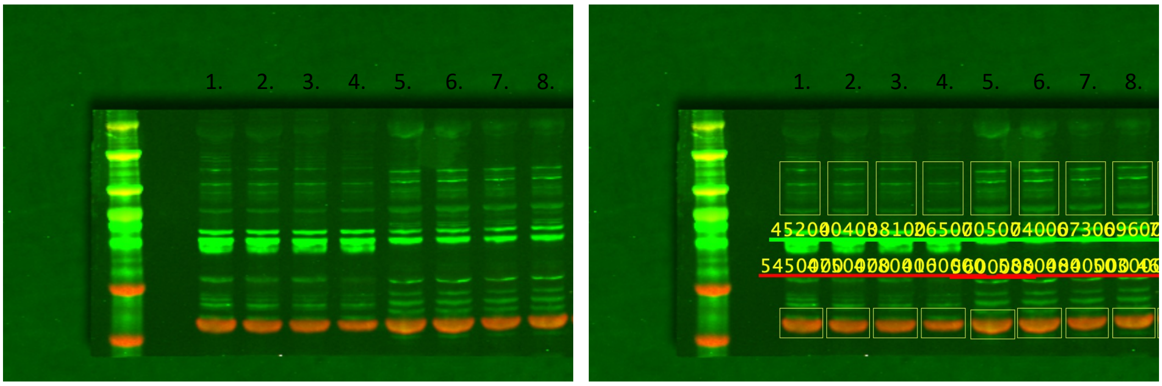

HIF1α Novus Lab Antibody (Figure S5)

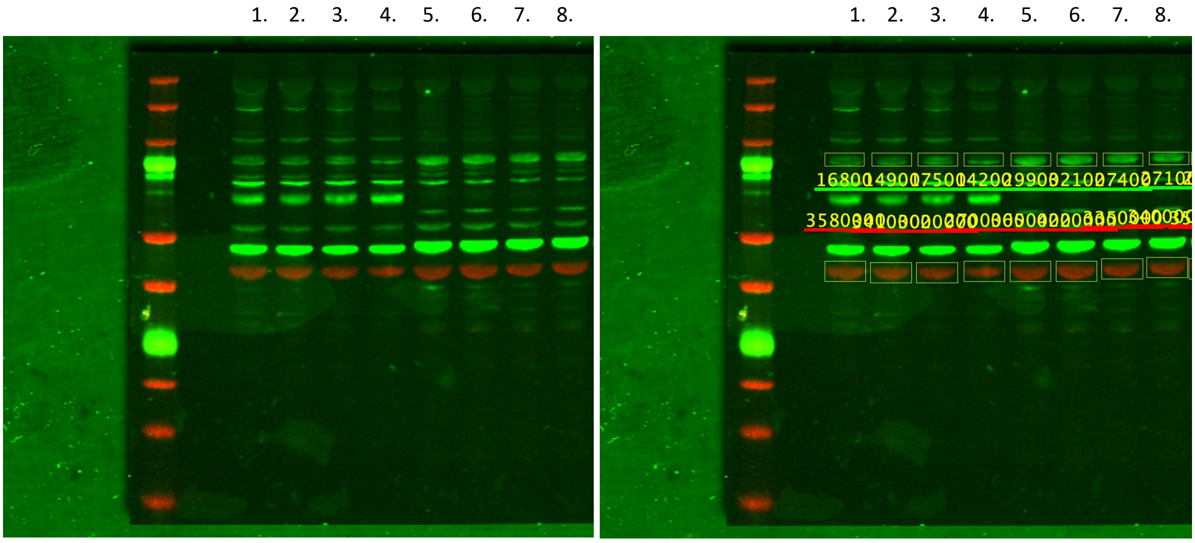

Supplement: Supplementary file 1 — Supplementary Information [file 41467_2024_46470_MOESM1_ESM.pdf]
