# Supplementary material for: Naked mole-rats have distinctive cardiometabolic and genetic adaptations to their underground low-oxygen lifestyles
Source: Nat Commun. 2024 Mar 27;15:2204. doi: 10.1038/s41467-024-46470-x (PMC10973426; doi:10.1038/s41467-024-46470-x)
Supplement: Supplementary file 3 — Description of Additional Supplementary Files [file 41467_2024_46470_MOESM3_ESM.pdf]

Description of Additional Supplementary Files

Supplementary Data 1

Excel file containing a normalised comparison of the 30 most expressed genes in the C57/BL6 mouse and all the subterranean species examined in this study. Genes are ranked in descending order according to FPKM values (Fragments Per Kilobase of transcript per Million mapped reads). Mole-rat and golden mole gene descriptors are based on the nearest description identified. Yellow filled cells are unique to the naked mole-rat top 30. Common colour fills represent shared genes.

Supplementary Data 2

Excel file containing a normalised comparison of the complete list of 20,285 expressed genes in the C57/BL6 mouse and the naked mole-rat heart. Gene expression is listed as FPKM values (Fragments Per Kilobase of transcript per Million mapped reads). Descriptors are based on the nearest gene description identified.

Supplementary Data 3

Excel file containing DEG data from RNA sequencing experiment (see supplementary methods above). Each sheet within the workbook shows pairwise DEGs between NMRs and each other subterranean species in the data set, together with Log2 fold changes, P-values and gene details.

Supplementary Data 4

Excel file containing normalised comparison of the complete list of expressed genes in the C57/BL6 mouse and the naked mole-rat heart and DEG analysis (Log2 fold change) of NMR-Mouse Orthologues. Gene expression is listed as FPKM (Fragments Per Kilobase of transcript per Million mapped reads).
