# Supplementary material for: Naked mole-rats have distinctive cardiometabolic and genetic adaptations to their underground low-oxygen lifestyles
Source: Nat Commun. 2024 Mar 27;15:2204. doi: 10.1038/s41467-024-46470-x (PMC10973426; doi:10.1038/s41467-024-46470-x)
Supplement: Supplementary file 8 — Reporting Summary [file 41467_2024_46470_MOESM8_ESM.pdf]

nature portfolio

Corresponding author(s): Dunja Aksentijevic

Last updated by author(s): 18/01/2024

Reporting Summary

Nature Portfolio wishes to improve the reproducibility of the work that we publish. This form provides structure for consistency and transparency in reporting. For further information on Nature Portfolio policies, see our Editorial Policies and the Editorial Policy Checklist.

Statistics

For all statistical analyses, confirm that the following items are present in the figure legend, table legend, main text, or Methods section.

n/a	Confirmed	
☐	☒	The exact sample size (*n*) for each experimental group/condition, given as a discrete number and unit of measurement
☐	☒	A statement on whether measurements were taken from distinct samples or whether the same sample was measured repeatedly
☐	☒	The statistical test(s) used AND whether they are one- or two-sided<br>*Only common tests should be described solely by name; describe more complex techniques in the Methods section.*
☐	☒	A description of all covariates tested
☐	☒	A description of any assumptions or corrections, such as tests of normality and adjustment for multiple comparisons
☐	☒	A full description of the statistical parameters including central tendency (e.g. means) or other basic estimates (e.g. regression coefficient) AND variation (e.g. standard deviation) or associated estimates of uncertainty (e.g. confidence intervals)
☐	☒	For null hypothesis testing, the test statistic (e.g. *F*, *t*, *r*) with confidence intervals, effect sizes, degrees of freedom and *P* value noted<br>*Give P values as exact values whenever suitable.*
☒	☐	For Bayesian analysis, information on the choice of priors and Markov chain Monte Carlo settings
☐	☒	For hierarchical and complex designs, identification of the appropriate level for tests and full reporting of outcomes
☒	☐	Estimates of effect sizes (e.g. Cohen's *d*, Pearson's *r*), indicating how they were calculated

Our web collection on statistics for biologists contains articles on many of the points above.

Software and code

Policy information about availability of computer code

Data collection	Ex vivo cardiac function: ADinstruments Chart v6<br>Cardiac histology Image J 1.53t<br>Hp Smart Colour Laser Scanner Software V14.1.0<br>Bruker TopSpin v4<br>ThermoFisher SCIENTIFIC XCell4 SureLock Midi Cell<br>Li-COR Odyssey Dlx Imaging System<br>Li-COR Imaging Studio Light Version 5.2.5
Data analysis	Graphpad Prism v9.0<br>ChenomX NMR profiler v 8.1<br>Bruker Topspin v 4<br>R studio: pheatmap, phyper, enhanced volcano plot<br>Microsoft Excel v16.77

For manuscripts utilizing custom algorithms or software that are central to the research but not yet described in published literature, software must be made available to editors and reviewers. We strongly encourage code deposition in a community repository (e.g. GitHub). See the Nature Portfolio guidelines for submitting code & software for further information.

Data

Policy information about availability of data

All manuscripts must include a data availability statement. This statement should provide the following information, where applicable:

- Accession codes, unique identifiers, or web links for publicly available datasets
- A description of any restrictions on data availability
- For clinical datasets or third party data, please ensure that the statement adheres to our policy

All the source data generated in this study have been deposited in the Dryad database under accession codes:
https://doi.org/10.5061/dryad.w9ghx3fts;
https://doi.org/10.5061/dryad.66t1g1k66
Source data files contain all data.
Heterocephalus glaber reference genome Ensemble_release-108

Research involving human participants, their data, or biological material

Policy information about studies with human participants or human data. See also policy information about sex, gender (identity/presentation), and sexual orientation and race, ethnicity and racism.

Reporting on sex and gender	Not applicable
Reporting on race, ethnicity, or other socially relevant groupings	Not applicable
Population characteristics	Not applicable
Recruitment	Not applicable
Ethics oversight	Not applicable

Note that full information on the approval of the study protocol must also be provided in the manuscript.

Field-specific reporting

Please select the one below that is the best fit for your research. If you are not sure, read the appropriate sections before making your selection.

☒ Life sciences ☐ Behavioural & social sciences ☐ Ecological, evolutionary & environmental sciences

For a reference copy of the document with all sections, see nature.com/documents/nr-reporting-summary-flat.pdf

Life sciences study design

All studies must disclose on these points even when the disclosure is negative.

Sample size	In all parts of the project we used the minimum number of animals per experiment that allowed robust, statistically and biologically significant results to be obtained. The number of animals purchase and maintained has been estimated based on the most cost-effective power calculations in the experimental models established in Aksentijevic laboratory. The number of mice required to find a statistically significant difference with 80% of power.
Data exclusions	No data was excluded
Replication	All analyses were done using at least 3 independent biological replicates/animals and repeated at least twice in separate experiments at different time points. All attempts at replication were successful.
Randomization	Samples were placed randomly into experimental groups.
Blinding	Analysis of all data was performed blinded to the phenotype.

Reporting for specific materials, systems and methods

We require information from authors about some types of materials, experimental systems and methods used in many studies. Here, indicate whether each material, system or method listed is relevant to your study. If you are not sure if a list item applies to your research, read the appropriate section before selecting a response.

Materials & experimental systems

n/a	Involved in the study
	x Antibodies
x	Eukaryotic cell lines
x	Palaeontology and archaeology
	x Animals and other organisms
x	Clinical data
x	Dual use research of concern
x	Plants

Methods

n/a	Involved in the study
x	ChIP-seq
x	Flow cytometry
x	MRI-based neuroimaging

Antibodies

Antibodies used

Proteintech rabbit polyclonal anti HIF-1α antibody (#: 20960-1-AP; Clone developed against HIF-1α fusion protein Ag15198 containing protein sequence including amino acids 574 - 799 of the human HIF-1 alpha protein; GenBank: BC012527.2). 1: 1000

Abcam rabbit monoclonal anti HIF-1α antibody (#: ab179483; Clone developed against recombinant fragment. This information is proprietary to Abcam and/or its suppliers). 1: 1000

Novus Lab rabbit polyclonal anti HIF-1α antibody (#: NB100-479; Clone developed against a fusion protein including amino acids 530 - 825 of the mouse HIF-1 alpha protein; Uniprot #Q61221). 1: 1000

ThermoFisher Scientific mouse monoclonal anti alpha Tubulin (#: 62204; IgG1 kappa developed against native chick brain microtubules, P09643-; PRID: AB_1965960). 1: 5000

Merck mouse monoclonal anti Actin antibody (#: MAB1501; Clone C4; IgG1 kappa developed against purified chicken gizzard actin). 1: 3000

Sigma Aldrich mouse monoclonal anti Vinculin (#: SAB4200729; Hybridoma VIN-11-5 clone; IgG1 developed against smooth muscle vinculin from chicken gizzard). 1: 5000

Abcam antibody cocktail (#: ab110413) contain mouse monoclonal antibodies against: CI subunit NDUFB8 (#: ab110242), CII-30kDa (#: ab14714), CIII-Core protein 2 (#: ab14745), CIV subunit I (#: ab14705) and CV alpha subunit (#: ab14748). 1: 1000

Abcam rabbit polyclonal anti Lactate Dehydrogenase (#: ab47010; IgG developed against synthetic peptide corresponding to Human Lactate Dehydrogenase aa 300 to the C-terminus conjugated to keyhole limpet haemocyanin). 1: 5000

Proteintech rabbit polyclonal anti MCT1 (#: 20139-1-AP; IgG developed against MCT1 Fusion Protein AG14098 including amino acid 451-500 encoded by BC026317; PRID: AB_2878645). 1: 1000

LI-COR goat anti rabbit IRDye 680RD coupled IgG Antibody (#: 926-68071; RRID AB_2721181). 1: 15000

LI-COR goat anti mouse IRDye 800RD coupled IgG Antibody (#: 926-32210; RRID RRID AB_2687825). 1: 15000

Validation

All antibodies have been validated by the manufacturers using cell treatment validation: detecting downstream events following the treatment, knockdown validation via the expression using RNAi to knock down the gene of interest, neutralization validation by functional blockage of protein activity by antibody binding, independent antibody verification: measurement of target expression is performed using two differentially raised antibodies recognizing the same protein target.

Animals and other research organisms

Policy information about studies involving animals; ARRIVE guidelines recommended for reporting animal research, and Sex and Gender in Research

Laboratory animals

NMRs were bred at the School of Biological and Behavioural Sciences, Queen Mary University of London. The non-breeding adult NMRs used in this study were second-generation or more captive-born, descended from animals captured in Kenya in the 1980s. Colonies were maintained using artificial burrow systems (group-housed in interconnected multi-cage systems) at 30°C and 21% O2 in 50% humidity with a 12h light cycle. Their diet consisted of fresh vegetables, fruit and tubers (sweet potatoes) ad libitum. All water requirements were obtained from the food resources.
C57/BL6 (J strain, 25g body weight, male) mice were purchased from Charles River Laboratories, UK and kept in individually ventilated cages, with access to food and water ad libitum, 12 hour light/dark cycle. Diet: PicoLab Mouse Diet 20 EXT (5R58), Product code I-DIET-5R58-9KG-BG supplied by IPS. Naked mole rats used in the study were 5 years old. C57/BL6 mice used in this study were 0.1 years.All mice within QMUL are housed in IVCs (Individual Ventilated Cages) and fed PicoLab mouse diet 5058 ad-lib. Enrichment is provided in the form of tunnels and chew sticks. Cages are cleaned out under a LEV hood when required, the maximum time between cage changes is 14 days.

Wild animals	Georychus capensis, Bathyergus suillus, Cryptomys hottentotus hottentotus, C. h. pretoriae, C. h. mahali, and C. h. natalenesis were wild-captured in South Africa using Hickman live traps, baited with a small piece of sweet potato. All traps were monitored for captures every 2-3 hours over the course of the day and left overnight, being checked first thing in the morning. Permission to capture these species was obtained from all landowners, and a collecting permit was obtained from the relevant nature conservation authorities (Permit number: Western Cape- CN44-87-13780, Gauteng- CPF6-0124, Kwa-Zulu Natal- OP1545/2021). Fukomys damarensis were laboratory maintained. Captured animals were brought back and individually housed at the University of Pretoria at ~27°C and 21% O2 in 50% humidity with a 12L:12D light cycle. All animals were housed in large polyurethane crates (1 × 0.5 × 0.5 m) with wood shavings and paper towelling for nesting material in temperature-controlled rooms. All wild-caught African mole-rat species were maintained in captivity for at least one year before tissue harvesting. Due to difficulty in maintaining the Golden moles in captivity and their short life-span, they were kept captive for approximately 3 weeks prior to tissue harvest. Each animal was terminally anaesthetised using isoflurane. Immediately upon cessation of breathing, heart was dissected and within 60 seconds placed in cryovial containing RNA later, and flash frozen in liquid nitrogen. Subsequently, samples were placed in the -80C freezer for storage. Age of animals: Cape mole=rat 5 years, cape mole=rat 5 years, cape dune mole-rat=4-5 years, common mole rat 4-5 years, highveld mole-rat=4-5 years, mahali mole-rat=4 years, natal mole-rat=4 years, damaraland mole-rat=6-7 years, hottentot golden-mole=adult >1years.
Reporting on sex	Sexes of mole rats were used in the study as summarised in Table S1.<br>Male only C56/BL6 mice were used as stated in the methods.
Field-collected samples	No field collected samples used in this study.
Ethics oversight	Our research complies with all the relevant ethical regulations. The Animal Use and Care Committee of the University of Pretoria evaluated and approved the experimental protocol and collection of all samples (ethics clearance number: NAS022/2021), with DAFF section 20 approval (SDAH-Epi-21051907211). Animal experiments at QMUL were approved by the local ethical review committee and in accordance with the Home Office Animals in Scientific Procedures Act 1986.

Note that full information on the approval of the study protocol must also be provided in the manuscript.
